# Prospective Study Evaluating Oncological Safety of Axillary Reverse Mapping

**DOI:** 10.1245/s10434-014-3626-5

**Published:** 2014-03-06

**Authors:** Eduardo Schunemann, Maíra Teixeira Dória, Janiceli Blanca Carlotto Hablich Silvestre, Plínio Gasperin, Teresa Cristina Santos Cavalcanti, Vinicius Milani Budel

**Affiliations:** 1 Department of Gynecology and Obstetrics, Clinical Hospital of Federal University of Paraná, Curitiba, Brazil; 2 South Paranaese Institute of Oncology, Ponta Grossa, Brazil; 3 Department of Pathology, Clinical Hospital of Federal University of Paraná, Curitiba, Brazil

## Abstract

**Background:**

Axillary reverse mapping (ARM) is a new technique developed with the aim of reducing lymphedema rates by preserving lymphatic drainage of the upper limbs during sentinel lymph node biopsy and axillary lymph node dissection (ALND). However, it is unclear whether preservation of these lymph nodes affects oncological risk. The present study evaluated the presence of metastases in ARM nodes.

**Methods:**

A total of 45 patients underwent ARM during ALND. Blue dye was used for ARM nodes localization. All axillary lymph nodes, including ARM nodes, were removed and sent separately for pathological evaluation of metastases.

**Results:**

ARM identification was achieved in 40/45 patients (88.9 %). The average number of removed ARM nodes was 1.9. ARM nodes metastasis occurred in 10 of 40 patients (25 %). Patients with an axilla extensively affected by cancer had an elevated risk of metastasis to the arm’s lymph nodes (*p* < 0.001).

**Conclusions:**

The rate of arm lymph nodes compromised by metastases calls into question the viability of the ARM technique. Larger studies may point to particular patient profiles for which ARM can be safely use.

Sentinel lymph node biopsy (SLNB) is currently the standard approach to determine breast cancer dissemination in patients with a clinical node-negative axilla. A randomized study by the American College of Surgeons’ Oncology Group, the Z0011 trial,^1^ recently showed that axillary lymph node dissection (ALND) can be omitted in patients with up to two positive sentinel lymph nodes (SLNs), calling into question how necessary ALND is in the treatment of breast cancer patients. However, the Z0011 trial included only patients at a T1 or T2N0M0 clinical stage with up to two positive SLNs treated with conservative surgery and radiation. ALND continues to be the standard treatment for patients not fitting the Z0011 trial criteria.^2^

ALND is associated with a higher rate of postoperative infection, seroma, lymphedema, paresthesia of the arm/axilla, and pain^3^,^4^ than SLNB alone. Notably, lymphedema has been reported to occur in 11–30 % of patients and is generally considered to be the most feared potential complication in these patients.^3^–^7^ SLNB has lower morbidity and lymphedema rates than ALND, although it is clinically significant at ~8 %.^3^

The technique of axillary reverse mapping (ARM) was developed in 2007 with the aim of reducing rates of lymphedema.^8^,^9^ The procedure is based on the hypothesis that the upper limb’s lymphatic drainage can be distinguished from the lymphatic drainage of the breast. Consequently, identification and preservation of the lymphatic arm drainage should result in avoidance of lymphedema. Initially, the involvement of ARM lymph nodes was not observed; however, there have since been reports of metastatic involvement of ARM lymph nodes and some concordance rate (crossover) between ARM nodes and the SLN.^10^–^18^ Thus, the oncological safety of this technique has been questioned. The present study was designed to evaluate the applicability of this technique and the presence of metastases in ARM nodes.

## Materials and Methods

### Patients

Between January 2010 and October 2012, we invited all women diagnosed with breast cancer and indicated for ALND (clinically node positive, clinically stage T3 tumors, and SLN compromised by macrometastases) who were treated at any of three treatment centers to participate in this study. The Ethics Committee at the Clinical Hospital of the Federal University of Paraná approved the study. A total of 45 patients signed informed consent forms and participated in the study.

### Procedure

Prior to skin incision, 1–5 ml of blue dye was injected into the subdermal area of the internal bicipital sulcus of arm ipsilateral to the breast with cancer. After massaging the area for 3 min, we proceeded with the standard surgical procedure planned for the patient. All axillary lymph nodes and the ARM node(s) were removed and sent separately to pathology.

### Pathological Examination

All nodes were entirely submitted for microscopic examination. They were cut into 2 mm slices in the longitudinal plane and when too thin they were submitted as one piece ‘in toto’. In the sequence they were processed, they were cut into microscopic slices of 4 μm and stained with hematoxylin and eosin (H&E). Histological diagnosis was made taking into account the American Joint Committee on Cancer (AJCC) Staging Manual, 7th edition.^19^

### Statistical Analyses

Data for the following characteristics were collected for each patient: age, tumor-node-metastasis (TNM) stage, histological tumor grade, tumor histology, estrogen receptor (ER) status, HER2/neu hyperexpression, use of neoadjuvant chemotherapy, type of surgery performed, pathologic staging of the axilla (TNM), number of upper-limb lymph nodes identified by ARM, and the presence of metastases in these lymph nodes. Mean values are reported with standard deviations (SDs).

A correlational analysis for the occurrence of metastases in the ARM nodes was conducted in relation to the following variables: age, neoadjuvant chemotherapy use, primary tumor histologic grade, and the axilla’s pathologic stage. The *χ* ^2^ test was used to reveal any associations between the variables. A *p* value <0.05 was considered significant.

## Results

ARM was performed on a total of 45 female breast cancer patients (mean age 49.4 years; range 35–85 years). The clinical characteristics of the patient cohort are summarized in Table 1. Briefly, the vast majority of patients had stage II or stage III cancer. The histologic grade of their primary tumors was most commonly grade II, followed closely by grade III, with grade I being relatively rare. Hormonal status was available in 41 of the 45 cases (91.1 %). Of these, a slight majority of the primary breast cancer tumors were ER positive. Sixteen patients showed elevated HER2/neu expression. A large majority of the patients underwent a modified radical mastectomy, whereas only a few had a skin-sparing mastectomy, and only one had a lumpectomy. A majority of the patients received neoadjuvant chemotherapy (60 %). Upper-limb lymph nodes were identified through ARM in 40/45 patients (88.9 %). All five patients in whom they could not be identified received neoadjuvant chemotherapy. The mean number of ARM nodes removed per patient was 1.9 (SD 1.8) and the mean number of axillary lymph nodes removed per patient was 15.5 (SD 8.0). 25 % (10/40) of patients had metastatic involvement of the ARM nodes.

**Table 1:** Clinical characteristics of the study cohort

Characteristic	No. of patients	%
Clinical T classification		
T1	2	4.5
T2	15	33.3
T3	18	40.0
T4	10	22.2
Clinical *N* classification		
N0	16	35.5
N1	26	57.8
N2	2	4.5
N3	1	2.2
Clinical stage		
I	2	4.5
IIA	6	13.3
IIB	14	31.1
IIIA	12	26.7
IIIB	10	22.2
IIIC	1	2.2
Histological grade		
1	6	13.3
2	21	46.7
3	18	40.0
Histology		
Ductal	40	88.9
Lobular	4	8.9
Not otherwise specified	1	2.2
ER status (*N* = 41)		
Positive	22	53.7
Negative	19	46.3
HER-2/neu (*N* = 41)		
Positive	16	39
Negative	25	61
Neoadjuvant chemotherapy		
Yes	27	60.0
No	18	40.0
Breast surgery		
Modified radical mastectomy	40	88.9
Skin-sparing mastectomy	4	8.9
Lumpectomy	1	2.2

*ER* Estrogen receptor

We evaluated the following variables in our analysis of metastatic ARM nodes: patient age, neoadjuvant chemotherapy use, histologic grade of the primary tumor, and number of lymph nodes in the axilla affected (pN0, pN1, pN2, and pN3). The resultant data are reported in Table 2. Briefly, patients with ARM nodes affected, as confirmed by *χ* ^2^ test, were, on average, significantly younger than patients without metastasis of ARM nodes. Of the ten cases with a metastatic upper-limb lymph node, nine were classified as pN2 or pN3, and one was classified as a pN1. Therefore, the number of positive axillary nodes was significantly associated with the involvement of ARM nodes (*p* < 0.001, *χ* ^2^ test). There was no significant association between metastatic involvement in ARM nodes and the histologic grade of the primary tumor (*p* = 0.342).

**Table 2:** Factors associated with metastasis-positive ARM nodes

Factor	No. of cases	ARM nodes		*p* value
		Positive	Negative	
Mean age, years (%)		44.1	52.2	0.015
Neoadjuvant chemotherapy [*n* (%)]				
Yes	22	7 (31.8)	15 (68.2)	NS
No	18	3 (16.7)	15 (83.3)	
Histologic grade [*n* (%)]				
1	6	2 (33.3)	4 (66.7)	
2	20	3 (15.0)	17 (85.0)	NS
3	14	5 (35.7)	9 (64.3)	
Axillary stage (pN, TNM) [*n* (%)]				
pN0	23	0 (0.0)	23 (100.0)	
pN1	6	1 (16.7)	5 (83.3)	
pN2 or pN3	11	9 (81.8)	2 (18.2)	<0.001

The five patients in whom ARM nodes could not be identified were excluded from these analyses

*ARM* Axillary reverse mapping, *NS* not significant

Of the 27 patients who received neoadjuvant chemotherapy, 7 had an upper-limb lymph node with metastases (25.9 %), whereas 3 (16.6 %) of the 18 patients who did not receive neoadjuvant treatment had metastatic ARM nodes. There was no statistical difference between these two groups (*p* = 0.464).

## Discussion

Questions remain regarding whether the identification rate for ARM nodes is satisfactory, whether the technique is safe, and whether preservation of ARM nodes reduces rates of lymphedema. The identification rate for arm-draining lymph nodes obtained in the present study (88.8 %) indicates that the ARM technique was effective in the great majority of patients and is in the upper range of rates reported in the literature (61.0–90.3 %) for ARM with patent blue dye^8^–^18^,^20^ (Table 3). Gennaro et al.^22^ injected an isotope on the back of the ipsilateral hand and were able to identify arm-draining lymph nodes in 45 of 60 patients (75 %). In a pilot study reported in 2009, Noguchi^23^ described a new technique in which they used a fluorescence imaging system with the injection of indocyanine green and were able to identify arm-draining lymph nodes in seven of eight patients. In a subsequent larger study published in 2012, Noguchi et al.^24^ achieved an identification rate of 85 % (29/34 patients) with this technique.

**Table 3:** Results of ARM during the ALND procedure

Reference	SLNB	No. of ARM procedures	Identification rates by ARM	Crossover rates	Metastatic involvement of ARM nodes	Technique
Thompson et al.^8^	No	18	61.0 %	0.0 %	0.0 % (0/7)	Blue dye
Nos et al.^9^	No	21	71.0 %	NR	0.0 % (0/10)	Blue dye
Nos et al.^21^	No	23	91.0 %	NR	14 % (3/21)	Blue dye and isotope
Casabona et al.^11^	Yes	9	88.9 %	0.0 %	0.0 % (0/3)	Blue dye
Kang et al.^10^	Yes	129	78.3 %	18.9 %	9 % (9/101)	Blue dye
Ponzone et al.^12^	No	49	73.5 %	NR	11 % (3/27)	Blue dye
Boneti et al.^17^	Yes	47	40.6 %	2.8 %	0.0 % (0/15)	Blue dye
Bedrosian et al.^13^	No	30	70.0 %	NR	13 % (2/15)	Blue dye
Deng et al.^14^	Yes	69	NR	8.7 %	8.7 % (6/69)	Blue dye
Rubio et al.^25^	No	36	83.3 %	14.0 %	13 % (4/30)	Blue dye
Gobardhan et al.^28^	No	93	90.3 %	NR	12 % (11/93)	Blue dye
Han et al.^15^	Yes	97	NR	7.2 %	12 % (2/17)	Blue dye
Noguchi et al.^24^	Yes	34	85.0 %	28.0 %	25 % (11/29)	Fluorescent
Tausch et al.^26^	No	143	78.0 %	NR	15 % (17/115)	Blue dye and isotope
Connor et al.^20^	Yes	57	72.0 %	10.0 %	15.7 % (3/19)	Blue dye
Gennaro et al.^22^	No	60	75.0 %	NR	No data	Isotope
Schunemann et al. (2014)	No	45	88.9 %	NR	25 % (10/45)	Blue dye

*ARM* Axillary reverse mapping, *SLNB* sentinel lymph node biopsy, *NR* not reported

The standard technique for identification of the SLN is the combined use of blue dye and isotope (technetium-99m). So far, only two authors used a combined technique to identify ARM nodes. In the study of Nos et al.,^21^ all patients underwent isotope injection in the ipsilateral hand the day before surgery, and they also had blue dye injection during anesthesia. These authors achieved an identification rate of 91 %. Tausch et al.^26^ performed ARM in 143 patients: 74 patients were injected with blue dye only; 8 patients were injected with radioactive only; and in 61 patients, a combination of blue dye and radioisotope was injected. The overall identification rate was 78 %: 62 % for blue dye only, 100 % for radioisotope only, and 95 % for the combined technique.

As summarized in Table 4, some authors have investigated whether the ARM technique can be applied during SLNB; in these cases, they obtained considerably lower identification rates for upper-limb lymph nodes (37.5–47.0 %) and sometimes considered the procedure insufficient.^10^,^11^,^14^,^15^,^17^,^18^,^20^,^23^ This difficulty arises mainly because of the location of the arm’s lymph node, which in most cases is situated below or at the level of the second intercostobrachial nerve, making it difficult to identify during SLNB.

**Table 4:** Summary of the literature results of ARM during SLNB

Reference	No. of ARM procedures	Identification rates	Crossover rates (%)	Metastatic involvement of ARM nodes	Technique
Boneti et al.^17^	131	42.7 %	3.9	0.0 % (0/12)	Blue dye
Casabona et al.^11^	72	37.5 %	0.0	0.0 % (0/3)	Blue dye
Kang et al.^10^	129	NR	18.9	8.3 % (8/96)	Blue dye
Boneti et al.^17^	220	40.6 %	2.8	0.0 % (0/15)	Blue dye
Deng et al.^14^	69	NR	8.7	8.7 % (6/69)	Blue dye
Han et al.^15^	14	NR	7.2	0.0 % (0/4)	Blue dye
Noguchi et al.^24^	97	43.0 %	28.0	11.9 % (5/42)	Fluorescent
Connor et al.^20^	155	47.0 %	12.0	0.0 % (0/18)	Blue dye

*ARM* Axillary reverse mapping, *SLNB* sentinel lymph node biopsy, *NR* not reported

The authors of the first studies published did not find metastases in the lymph nodes draining the arm, even in patients with an extensive nodal involvement.^8^,^9^,^17^ However, each of these studies included analyses of only a small number of ARM nodes. Thompson et al.^8^ performed the ARM procedure on 40 patients with an indication for SLNB with or without ALND; however, they sent only seven ARM nodes for pathological analysis. Similarly, Nos et al.^9^ included 21 patients indicated for ALND and managed to evaluate the ARM node in only ten cases. Boneti et al.^17^ performed ARM on 131 patients receiving SLNB, but only analyzed 12 ARM lymph nodes. Subsequently, Nos et al.^21^ were able to analyze nodes recovered from 21/23 patients indicated for ALND. Of the 21 recovered specimens, three had metastases (14 %). All three cases had an extensive compromised axilla (pN3a). More recently, other authors have found metastases in 8.7–25 % of ARM nodes.^8^–^14^,^18^,^21^,^24^–^26^ Since the series reported on thus far are small, it remains unclear how frequent ARM node involvement is and what the profile of at-risk patients might be.^27^

There are few studies that reported how the pathological examination of the ARM nodes was done. Gobardhan et al.^28^ examined the nodes after staining with H&E and immunohistochemically (IHC). As we did in our study, other authors^8^,^13^,^15^,^17^,^18^,^24^,^25^ analyzed the ARM nodes after staining with H&E. It is generally accepted that IHC are more sensitive for picking up micrometastasis compered with conventional H&E. However, we will probably not need to remove the ARM nodes with micrometastasis as we already do with the SLN with micrometastasis.

There are two possible explanations for metastatic involvement of arm-draining lymph nodes.^27^ First, it could be a consequence of the natural progression of the disease. There are lymphatic interconnections in the axilla between the arm and SNL draining from the breast. Breast cancer progression may alter the pattern of lymphatic flow, allowing the upper limb’s lymph nodes to be compromised. Second, it could be a result of the arm-draining lymph nodes being situated in the central group, which is too close to the breast’s lymphatic drainage to be preserved.

Special attention should be paid to convergence between the SLN and ARM nodes, which makes it impossible to preserve the arm-draining lymph nodes. Such convergence has been reported to occur in as little as 2.8 % and as much as 28 % of cases.^10^,^11^,^14^,^15^,^17^,^18^,^20^,^23^ In studies by Deng et al.^14^ and Noguchi,^23^ the ARM nodes with metastasis were the same as the SLN, showing that convergence is an important contributing factor to metastases in the lymph nodes of the upper limbs.^14^ However, in a study published by Rubio et al.,^25^ 4 of 30 patients (13 %) had metastases in ARM nodes, and none of the metastatic nodes corresponded with the SLN.

Our study did not evaluate convergence since all of our patients underwent ALND straightaway. Our rate of metastasis in the arm’s lymph nodes was higher than that found by Kang et al.^10^ (9 %), Deng et al.^14^ (8.7 %), and Han et al.^15^ (12 %). In these prior studies, the patient cohorts had a clinically negative axilla and therefore an indication for SLNB. They were early-stage patients with a low chance of axillary and ARM node involvement. We know that 40–60 % of patients with a positive SNLB finding do not have involvement of axillary lymph nodes beyond the SLN.^1^–^3^ Therefore, the rate of metastases in ARM nodes (25 %) in the present study is close to that of other studies in which only patients who already had an indication for ALND were included, such as the studies by Noguchi et al.^24^ (25 %) and Nos et al.^21^ (14 %).

Corroborating other previously published studies,^12^,^20^,^21^,^25^ our results show an association between the number of lymph nodes involved in the axilla and the presence of metastases in ARM nodes; patients with extensive involvement of cancer in the axilla are at greater risk of metastasis to the lymph nodes of the upper limbs. Thus, such patients should not be candidates for the ARM technique.

There are few studies that included patients who received neoadjuvant chemotherapy.^9^,^13^,^21^,^24^,^25^,^28^ Only our study and that of Gobardhan et al.^28^ evaluated the difference between the group receiving neoadjuvant chemotherapy versus a group that did not, and neither found a significant group difference for the incidence of metastasis in ARM nodes. Recently, studies have evaluated SLNB after neoadjuvant therapy in patients with an initially positive axilla.^29^–^31^ Both the ACOSOG Z1071 clinical study^30^ and the SENTINA study^31^ obtained higher-than-expected false negative rates (12.6 and 14.2 %, respectively). It would be premature to change standard clinical practices now. However, in the future, some patients with an initially positive axilla may be spared ALND if the SLNB technique can be proven reliable and oncologically safe after neoadjuvant chemotherapy.

The ARM technique was developed to preserve the arm’s lymph nodes during ALND and, consequently, to prevent lymphedema. However, very few studies have evaluated this outcome.^15^,^22^,^26^ Using ARM, Tausch et al.^26^ were able to preserve one or more upper-limb lymph nodes in 71/143 cases. After a median follow-up of 19 months, 35 of 114 patients developed lymphedema, and the ARM procedure was not associated with a significant reduction in morbidity. At the univariate analysis, obesity was the only risk factor for lymphedema. Gennaro et al.^22^ performed ARM on 60 patients, including 45 patients (group A) who underwent selective ALND wherein ARM nodes were preserved, and 15 patients (group B) who received standard ALND with removal of the arm’s lymph nodes. The two groups were similar in terms of risk factors for lymphedema. After monitoring for an average of 16 months, four patients in group A developed lymphedema (9 %) versus five patients in group B (33 %; *p* = 0.035). The number of patients in both of these studies^22^,^26^ was relatively small, as was the period of follow-up, which could lead to certain biases. Approximately 25 % of patients develop lymphedema more than 3 years after surgery.^5^ Thus, studies that monitor patients for less than 5 years are prone to underestimate the overall prevalence of lymphedema.^5^ Klompenhouwer et al.^32^ are currently carrying out a controlled, randomized study designed to investigate ARM’s ability to reduce the risk of lymphedema.

## Conclusions

Like other studies published to date, our study evaluated a small number of patients. However, the finding that 25 % of the ARM nodes had metastatic involvement is noteworthy. At this rate, it is oncologically unacceptable to preserve metastatic lymph nodes in the arm or those that coincide with the SLN during SLNB, which calls into question ARM’s viability and safety. Studies with a higher number of patients may be able to demonstrate whether there is a certain patient profile for which this technique could be applied safely.
